# Biomarkers of Cellular Stress Do Not Associate with sCD14 in Progressive HIV and SIV Infections *in Vivo*

**DOI:** 10.20411/pai.v5i1.363

**Published:** 2020-04-24

**Authors:** Carol L. Vinton, Carly E. Starke, Alexandra M. Ortiz, Stephen H. Lai, Jacob K. Flynn, Ornella Sortino, Kenneth Knox, Irini Sereti, Jason M. Brenchley

**Affiliations:** 1 Barrier Immunity Section; Laboratory of Viral Diseases; NIAID, NIH; Bethesda, Maryland; 2 HIV Pathogenesis Section; Laboratory of Immunoregulation; NIAID, NIH; Bethesda, Maryland; 3 Department of Medicine; University of Arizona; Tucson, Arizona

**Keywords:** Microbial translocation, HIV, SIV, inflammation

## Abstract

**Background:**

Microbial translocation occurs after damage to the structural and/or immunological barrier of the gastrointestinal (GI) tract into circulation. Microbial components that trans-locate from the lumen of the GI tract directly stimulate the immune system and contribute to inflammation. When microbial translocation becomes chronic, the inflammation has detrimental consequences. Given that microbial translocation is an important phenomenon in many diseases, defining biomarkers that reliably reflect microbial translocation is critical. Measurement of systemic microbial products is difficult since: 1) robust assays to measure microbial antigens simultaneously are lacking; 2) confounding factors influence assays used to detect microbial products; and 3) biological clearance mechanisms limit their detection in circulation. Thus, host proteins produced in response to microbial stimulation are used as surrogates for microbial translocation; however, many of these proteins are also produced in response to host proteins expressed by dying cells.

**Methods:**

We measured plasma levels of biomarkers associated with GI tract damage, immune responses to microbial products, and cell-death in people living with HIV before and after antiretroviral administration, and in macaque nonhuman primates before and after SIV infection.

**Results:**

Proteins secreted during cellular stress (receptor for advanced glycation endproducts-RAGE and high motility group box 1-HMGB1), which can induce sCD14 production *in vitro* and *in vivo*, do not associate with elevated levels of biomarkers associated with microbial translocation in progressively HIV-infected individuals and SIV-infected NHPs.

**Conclusions:**

Bystander cell death and generalized inflammation do not contribute to elevated levels of sCD14 observed in HIV/SIV-infected individuals.

## INTRODUCTION

Damage to the immunological and structural barrier of the gastrointestinal (GI) tract which occurs during acute HIV infection of humans and SIV infection of Asian macaques leads to translocation of microbial products from the lumen of the GI tract into circulation [1–3]. Significant loss of GI tract-resident CD4^+^ T cells [4], decreased production of IL-17 and IL-22 effector cytokines by GI tract-resident lymphocytes [5–7], alteration to the landscape of antigen presenting cells [5, 8], and epithelial cell apoptosis with loss of epithelial integrity [2, 9, 10] all contribute to epithelial barrier damage and, thus, microbial translocation in HIV/SIV infected individuals. Microbial products directly stimulate the immune system and, therefore, contribute to systemic inflammation in immunodeficiency lentiviral infections. While microbial translocation is one cause of systemic inflammation, the degree to which microbial translocation contributes to inflammation relative to HIV/SIV replication, and to frequently co-existing co-infections, in immunocom-promised individuals is not fully known. The contribution of HIV/SIV to inflammation can be assessed, to a large degree, by treating people living with HIV (PLWH) and SIV-infected NHPs with antiretroviral therapy (ART). Indeed, after administration of ART systemic inflammation decreases but remains elevated relative to healthy humans and SIV-uninfected NHPs. Residual inflammation observed in ART-treated PLWH and SIV-infected NHPs is associated with increased morbidity and mortality as compared to uninfected, population controls [11–14]. Thus, there is great interest in understanding the contribution of microbial translocation to this residual inflammation.

Measurement of the degree of microbial translocation occurring *in vivo* has been difficult. Bacterial DNA can be detected by quantitative PCR using primers which detect conserved sequences in the DNA that encode bacterial ribosomal RNA and elevated levels of bacterial DNA have been observed in PLWH [15–17]. The sensitivity of this assay is very low; however extracellular endonucleases in plasma and tissues can degrade extracellular DNA [18]. Additionally, bacterial DNA contamination is common in biomedical science which can confound the assay. The bacterial cell wall component lipopolysaccharide (LPS) can also be measured in biological materials by the limulus amebocyte lysate (LAL) assay. This assay takes advantage of an enzymatic reaction within the horseshoe crab (limulus) coagulation system that occurs in the presence of LPS. LPS levels are detectable in the pg/ml range with the LAL assay [1]. However, the ability of this assay to reliably detect LPS is greatly influenced by the presence of plasma proteins, such as high-density lipoproteins that result in its hepatic clearance and natural antibodies that recognize LPS, both of which can shield LPS from the LAL enzymatic reactions [19]. Thus, the ability to reliably measure systemic bacterial DNA and plasma LPS is highly variable.

A third approach to ascertaining levels of microbial translocation involves immunohistochemical staining for microbial products in paraffin-embedded tissue sections [2]. This requires tissues obtained at necropsy or after invasive biopsy procedures but allows for unambiguous determination of where microbial products reside, whether they co-locate with proinflammatory mediators, how effectively they are being cleared by phagocytes, and can be used to precisely enumerate products of microbial translocation with quantitative image analysis [2]. However, given the amount of tissue required, this approach is not easily adapted to large cohorts, or to longitudinal studies. Thus, while there is considerable interest in understanding the causes and consequences of microbial translocation, assays for precise quantitative determination remain undefined.

As a surrogate to measuring microbial products directly, many studies measure host proteins made, predominantly, in response to microbial antigenic stimulation. Commonly, plasma levels of soluble CD14 (sCD14, produced by myeloid cells after stimulation by LPS), lipopolysaccha-ride-binding protein (LBP, produced by hepatocytes after LPS-stimulation), and/or natural antibodies directed against LPS core antigen (EndoCAb) [20] are used as surrogate biomarkers. Of these, sCD14 is most commonly measured as an estimate of the level of microbial translocation given that it can be easily measured in plasma [20]. With TLR4, CD14 is part of the LPS receptor. Myeloid cells that are stimulated with LPS produce and secrete sCD14 and circulating sCD14 levels correspond to the degree to which myeloid cells were stimulated with LPS *in vivo* [21]. However, LPS is not the only ligand for CD14/TLR4 and the degree to which sCD14 levels accurately reflect microbial translocation in PLWH has been disputed [22–24]. RAGE shares common ligands and signaling pathways with the CD14/TLR4 complex - including high mobility group box protein 1 (HMGB1), a protein produced during cellular stress and a putative cause of chronic inflammation [25–27]. To understand the degree to which levels of HMGB1 and RAGE, proteins secreted during cellular stress, associate with sCD14 in PLWH and SIV-infected NHPs, here we measured plasma levels of RAGE, HMGB1, sCD14 and other markers associated with damage to the epithelial barrier of the GI tract such as intestinal fatty acid binding protein (IFABP) in cohorts of ART naïve and ART-treated PLWH and SIV-uninfected and SIV-infected NHPs. We find that neither HMGB1 nor RAGE are likely to be significant causes of elevated sCD14 levels observed routinely in progressively infected PLWH and SIV-infected NHPs.

## METHODS

### Study Subjects and Samples

Cohorts for this study consisted of longitudinal samples from 34 PLWH sampled prior to administration of ART and either one or four years after (Supplementary Table 1); 9 pigtail macaque monkeys (PTs) and 11 rhesus macaque monkeys (RMs) followed longitudinally pre-SIV infection and during acute (day 28 or 29 post-SIV infection) and chronic infection (day 197 or later post-SIV infection) (Supplementary Tables 2 and 3). All NHPs in this study were experimentally infected with SIVmac239.

All human study participants provided written informed consent prior to the study under institutional review board-approved protocol: Immune reconstitution syndrome in HIV-infected patients taking antiretroviral therapy (IRIS, NCT00286767) to NIAID, and protocol number 1011003018, Pulmonary CD4 T cell repopulation in immune reconstitution syndrome to the Indiana University.

All NHPs included in this study were housed and cared for in accordance with standards put forth by the American Association for the Accreditation of Laboratory Animal Care (AAALAC). All procedures were performed with approval and in accordance with the Institutional Animal Care and Use Committee within the National Institute of Allergy and Infectious Diseases (animal study protocol LVD26).

Plasma and peripheral blood mononuclear cells (PBMCs) were isolated from whole blood by standard density centrifugation and frozen (plasma) or cryopreserved (PBMC) so that all longitudinal study samples could be analyzed together.

### Measurement of Cellular Stress, Microbial Translocation, and Epithelial Damage Biomarkers

We measured plasma levels of HMGB1, sCD14, RAGE, IFABP, and zonulin by enzyme-linked immunosorbent assay (ELISA) in human and non-human primate samples (Table 1). All markers were analyzed according to the manufacturer's instructions.

**Table 1:** 

Analyte	Species	Company	Catalog	Plasma Dilution
HMGB1	Human, RM, PT	Antibodies Online	#abx151824	1:20
sCD14	Human, RM, PT	R&D Systems	#DC140	1:300
RAGE	Human	R&D Systems	#DRG00	undiluted
RAGE	RM, PT	My Biosource	#MBS746886	undiluted
IFABP	Human	Hycult	#HK406-02	1:4
IFABP	RM, PT	My Biosource	#MBS740424	undiluted
Zonulin	Human, RM, PT	Alpco	#30-ZONSHU-E01	1:20

### Plasma Viral Load and CD4 T Cell Assessment

SIV viral RNA levels in plasma were determined by real-time RT-PCR (ABI Prism 7900 sequence detection system; Applied Biosystems) using primer pairs corresponding to SIV_mac239_ gag gene sequences (forward, nucleotides 1181–1208, and reverse, 1338-1317). HIV viral RNA levels in plasma were determined by either a RTPCR COBAS 1.5 HIV Assay (Roche, with a lower limit of detection of 50 HIV copies/mL) or a RealTime HIV Assay (Abbott, with a lower limit of detection of 40 HIV copies/mL).

CD4^+^ T-cell counts were calculated from complete blood counts and CD4^+^ lymphocyte percentages determined via flow cytometric phenotypic profiling of PBMC samples from the same time-points.

## STATISTICAL ANALYSES

**Figure 1.**
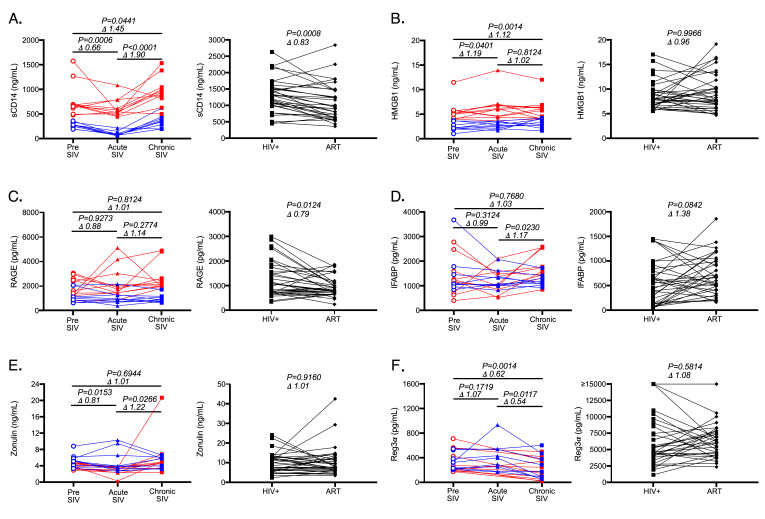
**Plasma biomarker levels in NHPs during SIV infection and in people living with HIV (PLWH) following ART.** (A-E) Plasma levels of biomarker analytes in NHPs (n = 20, graphs on the left, with PTs in blue and RMs in red) pre-SIV infection, during acute-SIV infection, and during chronic infection; and PLWH (n = 34, graphs on the right) pre- and post-ART treatment for (A) sCD14, (B) HMGB1, (C) RAGE, (D) IFABP, (E) Zonulin, and (F) Reg3α. Lines connect the same study participant over time. Wilcoxon t tests were used to determine each P-value. Δ values represent the mean fold-change between timepoints.

**Figure 2.**
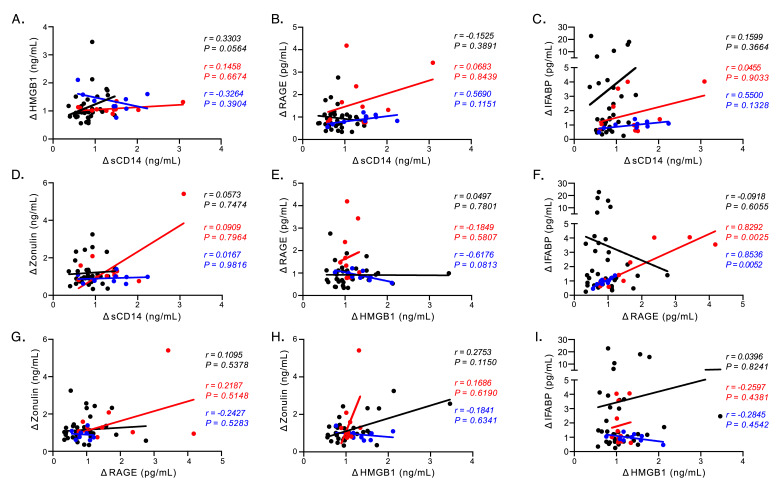
**Plasma biomarker associations.** (A-I) Correlational analyses between longitudinal changes in plasma biomarkers in humans (black), RMs (red), and PTs (blue). All associations were analyzed using the Spearman rank test.

**Figure 3.**
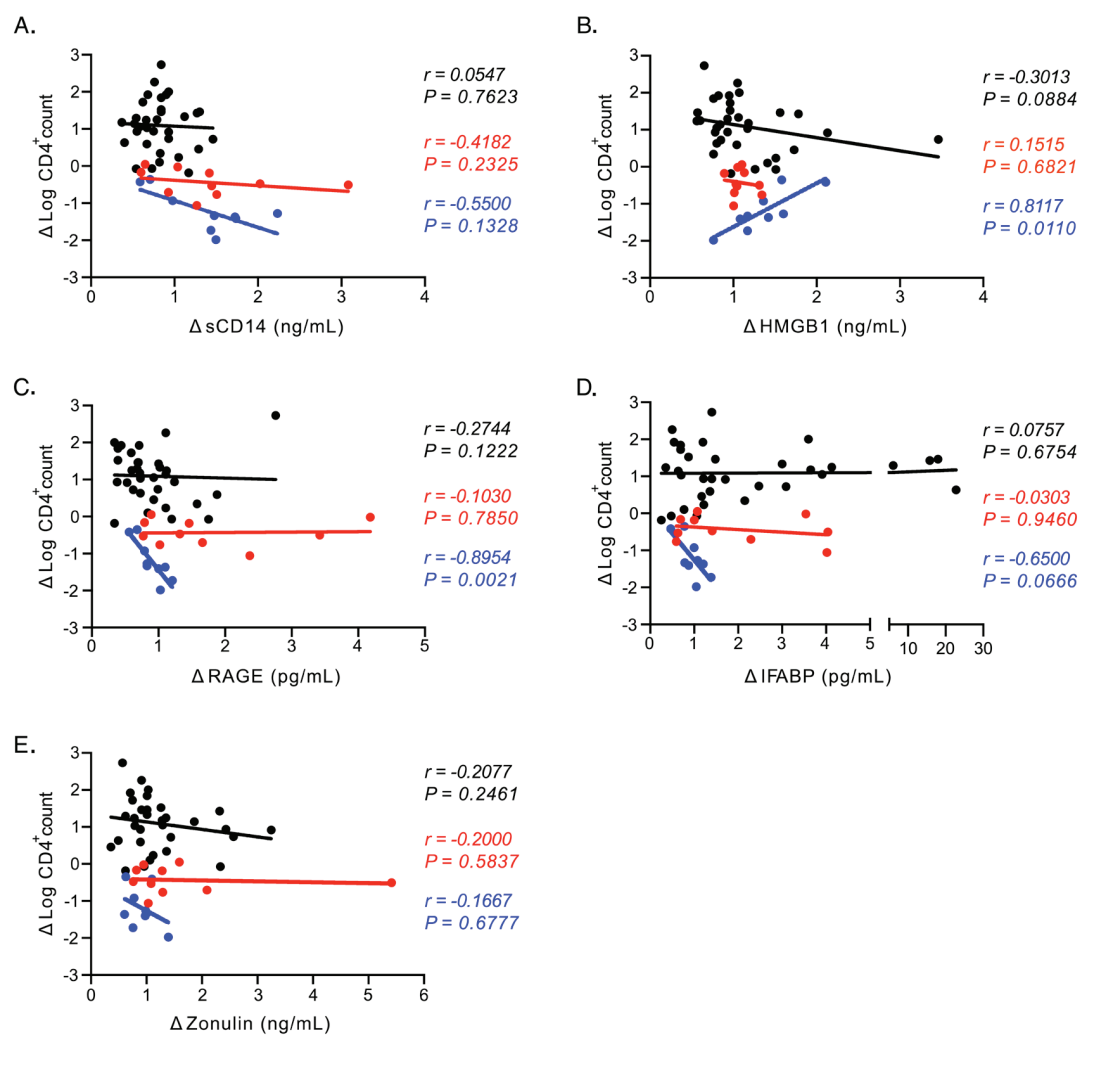
**Plasma biomarker associations with CD4 T cell frequencies.** (A-E) Correlational analyses between changes in CD4^+^ T cell numbers in peripheral blood and changes in plasma biomarkers in humans (black), RMs (red), and PTs (blue) for (A) sCD14, (B) HMGB1, (C) RAGE), (D) IFABP, and (E) Zonulin. All associations were analyzed using the Spearman rank test.

**Figure 4.**
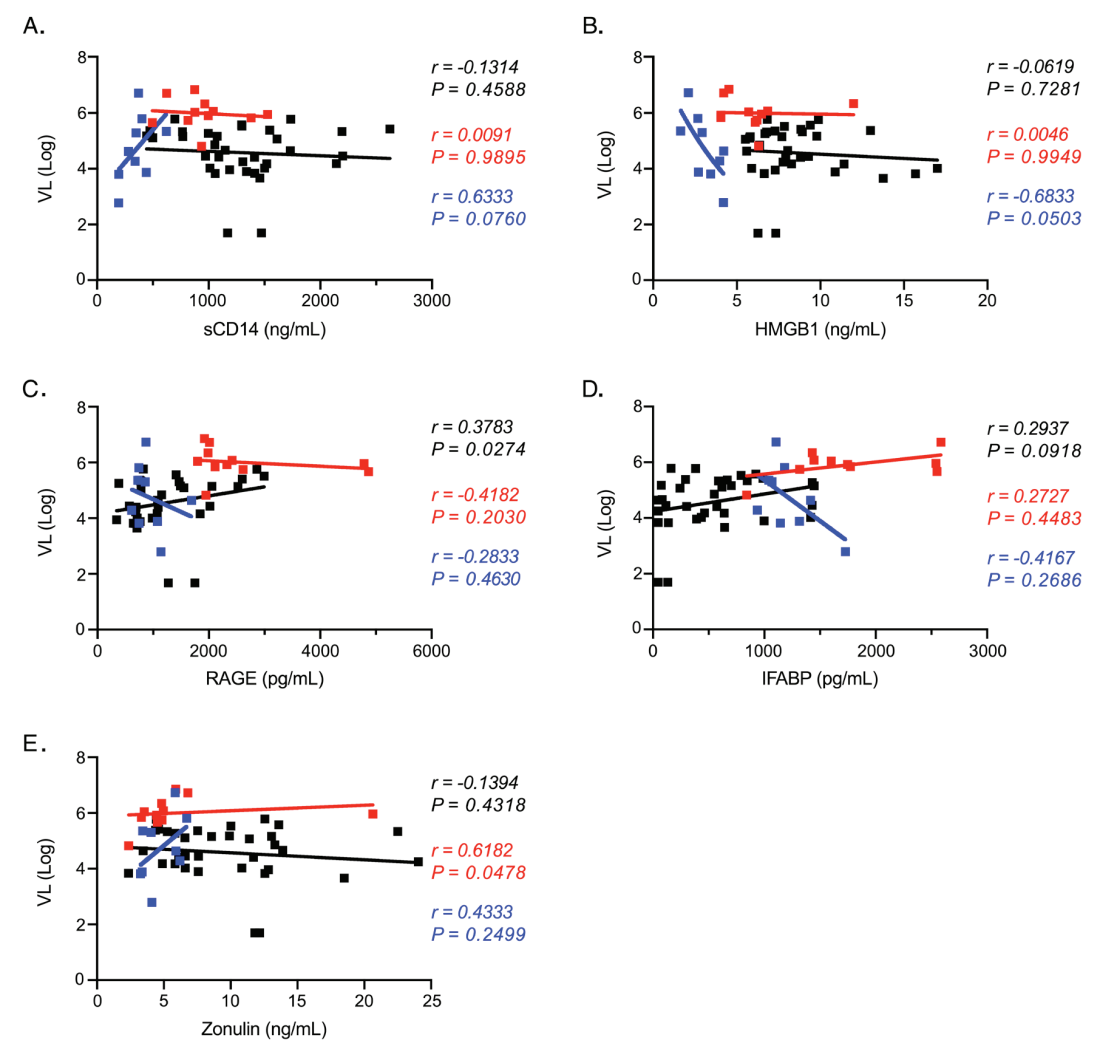
**Plasma biomarker associations with plasma viral load.** (A-E) Correlational analyses between SIV or HIV plasma viral load during chronic infection and plasma biomarkers in humans (black), RMs (red), and PTs (blue) for (A) sCD14, (B) HMGB1, (C) RAGE), (D) IFABP, and (E) Zonulin. All associations were analyzed using the Spearman rank test.

All statistical analyses were performed using Prism v8.0 (GraphPad Software). The Wilcoxon *t* test (matched pairs, two-way, nonparametric) was used for all analyses between longitudinal samples (Figure 1). All correlational analyses were performed using the Spearman rank test (Figures 2-4). Given correlations were measured in three different primate species, multiple comparison corrections were not considered.

## RESULTS

### Study Participant Cohorts

Here we assayed samples from PLWH pre-treatment and following ART (n=34) and Asian macaque NHPs prior to and throughout SIV-infection (total n = 20, 9 pigtailed macaques, PTs, and 11 rhesus macaques, RMs) (Supplementary Tables 1-3). PLWH had a mean CD4^+^ count of 75 cells/μl (range 0 to 444 cells/μl) prior to ART and exhibited a significant increase following ART (*P* < 0.0001, mean count = 385 cells/μl, range 149 to 759 cells/μl). The PLWH cohort had a mean plasma viral load of 127,698 copies (c)/mL (ranging from < 50 (2 patients) to 609,435 c/mL), with all of the study participants having viral loads below or close to the limit of detection after ART of at least 1 year (Supplemental Table 1).

SIV-infected cohorts had a mean acute plasma viral load of 584,338 c/mL (ranging from 1,736 to 2,403,815 c/mL) and mean chronic viral load of 1,267,104 c/mL (ranging from 621 to 7,200,000 c/mL). These SIV-infected cohorts demonstrated a decline in CD4^+^ T cell counts from pre-SIV infection through acute and chronic SIV-infection (with mean CD4^+^ T cell counts of 758 cells/μl for pre-, 325 cells/μl acute-, and 127 cells/μl chronic-SIV timepoints) (Supplemental Tables 2-3).

### Plasma Biomarker Levels

Consistent with several previous studies, we observed a marked increase in plasma sCD14 levels in SIV-infected NHPs from pre-SIV to chronic-SIV and from acute to chronic SIV-infection timepoints (Figure 1A, *P* = 0.04 and *P* < 0.0001) and significant decreases in the plasma levels of sCD14 in plasma of PLWH following antiretroviral treatment (ART, *P* = 0.0008, Figure 1A) [1, 3, 15]. Interestingly, sCD14 levels decreased during acute SIV infection (Figure 1A). While we have previously seen these levels to be moderately elevated during acute HIV infection [1], this apparent discrepancy is likely due to the well controlled longtidutinal analysis we were able to perform in NHPs. In order to determine if HMGB1 plasma levels followed similar patterns as sCD14 plasma levels during HIV/SIV infection and following ART, we next assayed this analyte in the same cohorts (Figure 1B). We found that HMGB1 levels were elevated from pre-SIV infection in both acute (*P* = 0.04) and chronic (*P* = 0.001) SIV infection timepoints; however, HMGB1 was not increased between acute and chronic SIV infection. In PLWH, plasma levels of HMGB1 demonstrated no significant consistent changes following ART (Figure 1B). Thus, alterations in the levels of sCD14 and HMGB1 were not consistent across SIV infection or after administration of ART in PLWH.

We next assessed levels of RAGE in plasma given its ability to induce sCD14 *in vitro* and *in vivo*. In Asian macaques levels of RAGE were entirely unaffected over the course of SIV infection (Figure 1C). In PLWH, RAGE levels significantly decreased after administration of ART (*P* = 0.01, Figure 1C). Thus, neither HMGB1 nor RAGE levels mirrored sCD14 level alterations which occur over the course of SIV infection of Asian macaques. However, RAGE levels in PLWH did follow a decreasing trend similar to that seen for sCD14 plasma levels after administration of ART in PLWH (*P* = 0.01, Figure 1C).

Microbial translocation occurs when there is a loss of/or damage to the GI tract epithelial barrier. Epithelial damage is thought to result in rapid release of epithelial fatty acid binding proteins and extracellular proteins involved in epithelial tight junctions into circulation [28, 29]. To assess the reliability of proteins released during epithelial damage as potential markers of microbial translocation, we measured plasma levels of IFABP and zonulin, a protein that functions to maintain intercellular junctions, in plasma. As Asian macaques progressed from acute to chronic infection, both IFABP (*P* = 0.02, Figure 1D) and zonulin (*P* = 0.03, Figure 1E) increased. In PLWH, 1-4 years of ART treatment was insufficient to result in significant decreases in these markers of intestinal damage (Figure 1D-E) consistent with previous reports [30]. In fact, IFABP levels actually tended to increase (*P* = 0.08, Figure 1D), likely due to increased epithelial cell turnover to repair the barrier. These data suggest that prolonged ART does not completely reduce GI tract damage observed in PLWH. Like HMGB1 and RAGE, these results did not track with sCD14 dynamics.

Another systemic biomarker which has recently been suggested to be associated with microbial translocation is Regenerating islet-derived protein 3α (Reg3α), an antimicrobial peptide produced by the GI tract epithelium [31]. Plasma levels of Reg3α have been shown to be elevated in chronic HIV infection and to correlate with plasma levels of lipopolysaccharide, pro-inflammatory cytokines, and percentages of activated CD4^+^ and CD8^+^ T cells [32]. Surprisingly, in our cohort of Asian macaques, plasma levels of Reg3α were unchanged during acute infection as compared to pre-infection levels, and actually decreased into chronic infection (*P* = 0.01, Figure 1F). Moreover, we found no significant differences in circulating plasma levels of Reg3α in our human cohort following ART (Figure 1F), while previous reports suggested these levels decrease in plasma of PLWH treated with ART [32].

### Biomarker Associations

Although markers of cellular stress, HMGB1 and RAGE, did not mirror sCD14 dynamics over the course of SIV infection or following ART in PLWH, we sought to determine if either analyte correlated with sCD14 or systemic levels of gut barrier damage. Positive associations would be supportive of their direct role in production of sCD14 *in vivo*. Even though HMGB1 levels were not decreased in PLWH treated with ART, changes over time in sCD14 trended towards a positive correlation with HMGB1 (human results in black r = 0.3303, *P* = 0.0564, Figure 2A). This association was not observed in either NHP species (RM results shown in red; PT results shown in blue Figure 2A). Interestingly, although longitudinal analysis of human plasma levels of RAGE trended consistently with sCD14 systemic plasma levels (Figure 1A and 1C), there were no significant correlations between changes in RAGE plasma level changes and longitudinal changes in sCD14 levels in either humans or NHPs (Figure 2B). sCD14 levels were not associated with IFABP or zonulin levels in any primate species (Figure 2C and 2D).

We next extended this analysis to determine if changes in HMGB1 or RAGE were associated with markers of GI tract damage. While HMGB1 and RAGE can both be elevated as a result of cellular damage, these were not associated with one another in any of the primate species we studied (Figure 2E). We did find strong associations between changes in plasma levels of RAGE and IFABP in both NHP groups (RM r = 0.83 and *P* = 0.0025; PT r = 0.85 and *P* = 0.0052, Figure 2F) with no such association being observed in our human cohort. However, changes in RAGE did not associate with change in zonulin levels in either NHP cohort (Figure 2G). Furthermore, changes in HMGB1 plasma levels did not correlate with either of the markers of epithelial damage we assayed (zonulin Figure 2H and IFABP Figure 2I). From these analyses, the data suggest that in progressive HIV/SIV infections neither HMGB1 nor RAGE contribute to elevated levels of biomarkers used as surrogates for microbial translocation.

### Biomarker Associations with Canonical Measures of HIV/SIV Disease Progression

We next determined whether canonical measures of HIV/SIV disease progression were associated with levels of any plasma biomarkers we studied. In particular, we studied associations with changes in CD4^+^ T cell counts (Figure 3) and changes in plasma viral load (Figure 4) over time. For PLWH we looked at the change in value from chronic infection to post-ART (Δ ART/chronic baseline), while for NHPs we looked at the change in values from pre- to chronic SIV-infection (Δ chronic/pre). Among the five studied biomarkers, only HMGB1 (Figure 3B) and RAGE (Figure 3C) showed correlations with changes in the numbers of CD4^+^ T cells in peripheral blood; however these associations were seen only in the PT cohort- no other correlations were observed.

Lastly, direct or indirect effects of viral components (such as viral RNA) can be directly stimulatory to the immune system, thus alternately explaining elevated levels of sCD14 and other biomarkers used as surrogates for microbial translocation [33–35]. Therefore, we measured relationships between plasma viremia and each of the biomarkers we studied during chronic infection (Figure 4). In PTs HMGB1 was actually negatively correlated with plasma viremia (Figure 4B), while in humans RAGE levels positively correlated (Figure 4C). However, these associations were not observed across all three primate species and we found no associations between plasma viremia and either sCD14 (Figure 4A) or IFABP (Figure 4D). These data argue against plasma viremia (either directly or indirectly) leading to altered levels of these biomarkers.

## DISCUSSION

Microbial translocation is a well described phenomenon in chronically infected PLWH and SIV-infected Asian macaques [1, 3, 20]. Even after decades of ART - particularly if ART was initiated when nadir CD4^+^ T cell counts in peripheral blood were low - the damage that had occurred to the GI tract is not completely reversed, [30, 36, 37]. Residual inflammation which occurs in these treated individuals leads to increased mortality from different malignancies and cardiovascular disease and may be attributed, at least partially, to ongoing microbial translocation [11, 30, 38, 39]. While the current World Health Organization's recommendations are to initiate therapy as early as possible in all PLWH [40], most PLWH currently taking ART did not initiate therapy until the chronic phase of infection. These individuals may benefit from supplemental therapies which restore the GI tract and reduce microbial translocation and inflammation. Thus, there is a great need to identify putative biomarkers which can be used to monitor efficacy of strategies aimed at reducing microbial translocation and inflammation. Of the numerous plasma analytes which might fulfill this purpose, sCD14 is, by far, most frequently studied. Although sCD14 levels represent bioactivity of microbial products, several studies have shown that sCD14 levels can be elevated in the apparent absence of microbial translocation [22, 24]. While CD14 is, indeed, a specific receptor for LPS, HMGB1 is also a ligand for CD14 [25–27].

Here we sought to determine how levels of particular plasma biomarkers associated with microbial translocation and inflammation change over the course of SIV infection of NHPs and after administration of ARTs in HIV-1-infected individuals. We also sought to determine whether HMGB1 and RAGE might contribute to the elevated plasma levels of sCD14 observed in progressive HIV/SIV infections. Our data suggest that HMGB1 and RAGE are not associated with elevated levels of sCD14 observed in PLWH and SIV-infected NHPs given that neither HMGB1 nor RAGE levels reliably changed over time as NHPs became SIV-infected or PLWH initiated ARTs. Thus, elevated sCD14 is unlikely to be attributed to cellular death in these cases. We also do not find evidence that markers of intestinal epithelial damage correlate with sCD14, suggesting that these epithelial markers themselves are not sufficient biomarkers of microbial translocation and epithelial turnover after administration of ART could contribute to elevated levels of IFABP.

There is significant interest in understanding the mechanisms underlying microbial translocation, the degree to which microbial translocation contributes to inflammation observed in PLWH, and in developing therapeutic interventions which ameliorate microbial translocation [3, 41–43]. Assays to detect bacterial products in plasma lack sensitivity and reproducibility and assays to measure bacterial products in tissue require invasive procedures. While our study included a small cohort of NHPs and PLWH, and would have benefitted from inclusion of tissue biopsies, we believe our study demonstrates that measurement of sCD14 in plasma in conjunction with proteins which are released into circulation when GI tract epithelial cells are damaged (IFABP and zonulin) are currently the best available surrogates of systemic microbial translocation. We also believe that tissue measurement of microbial products remains the most robust measurement of microbial translocation. As next generation sequencing approaches become more available and accessible, the ability to detect nucleic acids corresponding to microbes in circulation may alleviate these issues [44].

## SUPPLEMENTARY MATERIALS

**Supplementary Table 1:** Study Participants – PWH

ID	Timepoint	Plasma Viremia^a^	CD4^+^ count^b^	Sex	Age	Race/Ethnicity	Tobacco Use	Albumin Levels^c^
1	HIV+ ARV−	6,810	0	Female	25	African American	No	3.5
	HIV+ ARV+ (4 yrs)	<40	542		29			4.0
2	HIV+ ARV−	26,202	45	Female	37	African American	No	3.0
	HIV+ ARV+ (4 yrs)	<40	510		41			4.2
3	HIV+ ARV−	266,478	35	Male	41	Multiple Races/Hispanic or Latino	No	3.9
	HIV+ ARV+ (4 yrs)	<50	298		45			3.7
4	HIV+ ARV−	28,432	33	Male	39	African American	No	3.8
	HIV+ ARV+ (4 yrs)	<40	571		43			4.3
5	HIV+ ARV−	6,842	12	Female	41	African American	No	2.8
	HIV+ ARV+ (4 yrs)	<50	322		45			3.6
6	HIV+ ARV−	116,716	44	Male	53	African American	Yes	3.3
	HIV+ ARV+ (4 yrs)	<40	241		57			3.3
7	HIV+ ARV−	<50	82	Male	25	African American	Yes	3.7
	HIV+ ARV+ (4 yrs)	<40	353		29			4.1
8	HIV+ ARV−	601,301	5	Male	27	Caucasian/Hispanic or Latino	No	2.7
	HIV+ ARV+ (4 yrs)	<40	347		31			4.3
9	HIV+ ARV−	240,966	43	Female	32	African American	No	4.0
	HIV+ ARV+ (4 yrs)	<40	759		36			3.7
10	HIV+ ARV−	231,414	3	Male	31	African American	No	3.0
	HIV+ ARV+ (4 yrs)	<40	547		36			4.0
11	HIV+ ARV−	25,831	16	Male	31	Caucasian/Hispanic or Latino	Yes	3.4
	HIV+ ARV+ (4 yrs)	60	172		35			3.7
12	HIV+ ARV−	28,342	6	Female	35	African American	No	2.5
	HIV+ ARV+ (4 yrs)	<40	605		39			3.8
13	HIV+ ARV−	186,674	231	Male	40	Caucasian/Hispanic or Latino	No	2.2
	HIV+ ARV+ (4 yrs)	<40	195		44			4.6
14	HIV+ ARV−	379,425	7	Female	25	Multiple Races/Hispanic or Latino	No	3.1
	HIV+ ARV+ (4 yrs)	<40	588		29			4.1
15	HIV+ ARV−	149,655	45	Male	46	Caucasian/Hispanic or Latino	Yes	4.0
	HIV+ ARV+ (4 yrs)	<40	238		50			4.2
16	HIV+ ARV−	145,610	18	Female	46	Caucasian/Hispanic or Latino	No	3.9
	HIV+ ARV+ (4 yrs)	<40	149		50			4.6
17	HIV+ ARV−	10,603	12	Male	37	Multiple Races/Hispanic or Latino	No	2.6
	HIV+ ARV+ (4 yrs)	<50	167		41			3.6
18	HIV+ ARV−	<50	18	Female	31	Caucasian/Hispanic or Latino	No	2.6
	HIV+ ARV+ (4 yrs)	<40	352		35			3.9
19	HIV+ ARV−	9,251	74	Female	55	African American	No	3.6
	HIV+ ARV+ (4 yrs)	<40	291		59			3.9
20	HIV+ ARV−	142,379	11	Male	41	Caucasian/Hispanic or Latino	No	4.0
	HIV+ ARV+ (4 yrs)	<40	368		45			4.4
21	HIV+ ARV−	128,742	12	Male	41	African American	No	3.4
	HIV+ ARV+ (4 yrs)	<50	258		45			4.0
22	HIV+ ARV−	212,286	74	Male	34	Unknown/Hispanic or Latino	Yes	3.4
	HIV+ ARV+ (4 yrs)	<50	644		39			4.1
23	HIV+ ARV−	339,630	12	Female	32	Caucasian/Hispanic or Latino	No	3.7
	HIV+ ARV+ (4 yrs)	<40	630		36			4.2
24	HIV+ ARV−	71,873	6	Female	33	African American	No	3.4
	HIV+ ARV+ (4 yrs)	<40	503		37			3.9
25	HIV+ ARV−	609,435	124	Male	48	African American	No	3.6
	HIV+ ARV+ (4 yrs)	<40	212		52			4.0
26	HIV+ ARV−	45,764	24	Male	35	Caucasian/Hispanic or Latino	No	3.7
	HIV+ ARV+ (4 yrs)	<40	361		39			4.3
27	HIV+ ARV−	43,907	12	Female	29	African American	Yes	3.7
	HIV+ ARV+ (4 yrs)	<40	349		33			4.1
28	HIV+ ARV−	15,269	12	Female	43	African American	No	4.2
	HIV+ ARV+ (4 yrs)	<40	347		47			4.3
29	HIV+ ARV−	15,000	362	Male	24	Caucasian	Yes	ND
	HIV+ ARV+ (1 yr)	<50	457		25			ND
30	HIV+ ARV−	10,600	182	Female	47	African American	Yes	ND
	HIV+ ARV+ (1 yr)	<400	400		48			ND
31	HIV+ ARV−	4,620	444	Female	49	African American	Yes	ND
	HIV+ ARV+ (1 yr)	<50	291		50			ND
32	HIV+ ARV−	7,870	372	Male	34	African American	Unknown	ND
	HIV+ ARV+ (1 yr)	<50	319		35			ND
33	HIV+ ARV−	216,000	113	Male	Unknown	Caucasian	No	ND
	HIV+ ARV+ (1 yr)	<48	323					ND
34	HIV+ ARV−	17,700	45	Female	34	African American	Yes	ND
	HIV+ ARV+ (1 yr)	ND	ND		35			ND

a Plasma viral load (copies/mL)

b CD4+ T cell count (cells/ul blood)

c Plasma albumin levels (g/dL)

**Supplementary Table 2:** Study Participants – Pigtail Macaques

ID	Timepoint	Plasma Viremia^a^	CD4+ count^b^	Sex	Age	Albumin Levels^c^
PT98P030	Pre-SIV	0	976	Female		
	Acute SIV	1,736	ND			
	Chronic SIV	621	372		11.5	ND
PTA0P039	Pre-SIV	0	1,144	Female		
	Acute SIV	1,767,348	230			
	Chronic SIV	19,161	45		9.5	3.6
PT99P052	Pre-SIV	0	1,597	Female		
	Acute SIV	331,131	ND			
	Chronic SIV	643,458	30		10.5	3.2
PT99P029	Pre-SIV	0	765	Female		
	Acute SIV	475,587	355			
	Chronic SIV	5,394,895	8		10.8	2.3
PT98P005	Pre-SIV	0	765	Female		
	Acute SIV	323,594	203			
	Chronic SIV	7,625	33		12	2.1
PT99P030	Pre-SIV	0	842	Female		
	Acute SIV	103558	369			
	Chronic SIV	6,600	372		11.2	3.8
PTA0P007	Pre-SIV	0	653	Female		
	Acute SIV	125,440	199			
	Chronic SIV	43,311	31		10	3.5
PTA1P012	Pre-SIV	0	1,347	Female		
	Acute SIV	355,277	387			
	Chronic SIV	201,583	160		9.2	4.1
PTA0P012	Pre-SIV	0	690	Female		
	Acute SIV	2,403,815	154			
	Chronic SIV	226,244	37		19.9	4

a Plasma viral load (copies/mL)

b CD4+ T cell count (cells/ul blood)

c Plasma albumin levels at chronic timepoint (g/dL)

ND = not done

**Supplementary Table 3:** Study Participants – Rhesus macaques

ID	Timepoint	Plasma Viremia^a^	CD4+ count^b^	Sex	Age	Albumin Levels^c^
RHCL4C	Pre-SIV	0	158	Male		
	Acute SIV	873,043	219			
	Chronic SIV	840,000	47		14	2.8
RHCL7P	Pre-SIV	0	886	Male		
	Acute SIV	612,776	1,012			
	Chronic SIV	560,000	ND		14.3	3.6
RHCL86	Pre-SIV	0	1,037	Male		
	Acute SIV	1,471,247	251			
	Chronic SIV	1,100,000	91		14	3.4
RHDCBC	Pre-SIV	0	446	Male		
	Acute SIV	133,266	454			
	Chronic SIV	1,200,000	ND		8.4	2.8
RHDCJWA	Pre-SIV	0	730	Male		
	Acute SIV	556,021	195			
	Chronic SIV	700,000	ND		8.3	2.6
RHDCKJ	Pre-SIV	0	966	Male		
	Acute SIV	627,870	853			
	Chronic SIV	5,300,000	192		8.1	3.8
RHDCVF	Pre-SIV	0	410	Male		
	Acute SIV	178,242	160			
	Chronic SIV	7,200,000	268		7	3.5
RHDE1A	Pre-SIV	0	339	Male		
	Acute SIV	557,837	182			
	Chronic SIV	200,000	ND		7.8	3.4
RHDE20	Pre-SIV	0	747	Male		
	Acute SIV	709,493	304			
	Chronic SIV	67,000	ND		7.5	3.6
RHDE2C	Pre-SIV	0	454	Male		
	Acute SIV	12,688	154			
	Chronic SIV	470,000	ND		7.4	3.4
RHDE2W	Pre-SIV	0	297	Male		
	Acute SIV	66,799	165			
	Chronic SIV	920,000	93		7	2.1

a Plasma viral load (copies/mL)

b CD4+ T cell count (cells/ul blood)

c Plasma albumin levels at chronic timepoint (g/dL)

ND = not done
